# Acute Sinusitis in Children - A retrospective study of orbital complications

**DOI:** 10.1016/S1808-8694(15)31126-5

**Published:** 2015-10-20

**Authors:** Levon Mekhitarian Neto, Shirley Pignatari, Sérgio Mitsuda, Antonio Sérgio Fava, Aldo Stamm

**Affiliations:** 1 MS. Student; 2 Assistant Professor - Pediatric ENT Discipline - UNIFESP; 3 MD. Otorhinolaryngologist - Prof. Edmundo Vasconcelos Hospital; 4 Professor at the Postgraduate Course - Heliópolis Hospital; 5 PhD in Otorhinolaryngology - UNIFESP

**Keywords:** children, orbital, cellulitis, complications, sinusitis

## Abstract

**Summary:**

Among the complications of sinusitis, those that involve the orbital region are the most frequent.

**Aim:**

The objective of this paper is to show an incidence of orbital cellulites (OC) secondary to acute sinusitis in children. Study design: retrospective.

**Methods:**

After board approval, the charts of all pediatric patients diagnosed with orbital complications secondary to sinusitis, seen at the Pediatric and at the ENT clinics of the HPEV, between 1985 and 2004, were evaluated. The data was analyzed considering gender, age, clinical presentation, period of hospitalization, image study, and treatment.

**Results:**

from 1985 to 2004, 25 patients were diagnosed with OC secondary to sinusitis, presenting an incidence of 6%. Males predominated, the median age was 6.5 years, and the maxillary was the most frequently involved sinus. Twenty-four patients presented mild periorbital swallowing. All 25 patients presented X-Ray alterations. One patient with proptosis had a subperiosteal abcess seen on the CT-scan. The average time of hospitalization was 4 days. All 25 patients received IV antibiotics, 2 required surgery.

**Conclusion:**

The incidence of orbital complications secondary to sinusitis is low, and although the majority of cases are early diagnosed and respond well to medication treatment, a surgical intervention may be required.

## INTRODUCTION

Bacterial infection in the paranasal sinuses is one of the most frequent diseases both in adults and children. Almost always secondary to a viral infection of the upper airways, sinusitis usually brings about rhinorrhea, nasal obstruction, headache and fever, among other signs and symptoms.

Among sinusitis's complications, the ones that involve the eye orbit region are the most frequent and usually associated ethmoid sinuses involvement in younger patients^1^, ^2^, ^3^, ^4^, ^5^, ^6^, ^7^. This is mainly caused by the close anatomical relations between the eye orbit cavity and the ethmoid sinuses, as well as to the frailty of the ethmoid-orbit wall, especially in children^8^,^9^. It is estimated that sinusitis complications before the time of antibiotics occurred in 1 for each 5 patients, and the rates of both morbidity and mortality related to orbitary cellulites were very high; from 17 to 20% of these patients died because of meningitis or ended up with some permanent visual impairment in the affected eye^10^, ^11^, ^12^, ^13^. Currently, these sequels do not reach 5% of the cases.

Since sinusitis was not easily diagnosed before the time of antibiotics, orbitary sinusitis was associated to sinus problems^10^. Today, it is believed that this ratio is of about 70% and that 60 to 80% of orbitary inflammatory diseases come from the sinuses. They are considered severe situations, and in some instances treatment must include surgical drainage of the pus in the affected paranasal sinus.^8^,^9^,^13^, ^14^, ^15^.

Although today post-sinusitis orbitary complications are less common, especially because of the ease with which we have image studies that allow for a more accurate and early diagnosis, and the use of large spectrum antibiotic agents that aid in the proper treatment of the infectious processes, these situations keep on occurring and bear the same severity if not proper diagnosed and treated.

Periorbitary venous drainage is carried out by valveless veins that interconnect the paranasal sinuses with the orbit, the cavernous sinus and the facial tissue^10^,^12^,^16^. This free system of anastomosis allows phlebitis and thrombophlebitis to progress and invade these adjacent structures^17^.

Most clinical investigators call orbitary cellulitis any orbit complication with or without pus, classifying the cases according to severity and the extension of the infectious process^18^,^19^.

In 1948 Smith and Spencer^20^ introduced a classification for sinusitis complications used in a series of adult patients, emphasizing that the categories would simple mean an artificial division of a continuous process, staging this process according to disease severity. Chandler et al., in 1970, modified this classification, and since then it has been used internationally^21^,^22^.

In all classifications proposed, proptosis seems to be the finding that differentiates the most severe stages of the complication. Proptosis may also be used as a guide to help locate the abscess. If the proptosis is symmetrical, there is an even greater chance of orbit content involvement. However, if asymmetrical, the abscess may be located in the opposite orbitary quadrant. In general, the larger the proptosis, more severe is the inflammation or the abscess size^21^.

In those patients who developed orbitary cellulitis, 10% have temporary visual loss in the affected eye^23^. Other complications may occur less frequently and include meningitis, frontal osteomyelitis, intracranial abscess, etc.

The treatment of these complications require a team of different specialists, the otorhinolaryngologist taking care of the sinus infection, the ophthalmologist tending to the visual complications and the pediatrician taking care of the clinical problems. Clinical treatment is based on the use of high doses of intravenous antibiotics capable of crossing the blood-brain barrier, and by monitoring the response through systemic and visual signs and symptoms^11^. According to Lusk^12^, if the edema progresses, there is loss of visual acuity and/or a fast decline in clinical status, one should order a CT Scan or MRI. Should an abscess be seen, there is the need for immediate surgical intervention, by conventional means or functional endoscopic approach.

The goal of the present study is to show the incidence of orbitary cellulitis as a complication of acute sinusitis in children admitted to the pediatrics and otorhinolaryngology wards of the Prof. Edmundo Vasconcelos hospital, classifying the clinical picture (Chandler's classification) and the treatment installed.

## MATERIALS AND METHODS

The present investigation was based on the retrospective analysis of the charts of patients admitted to the Pediatrics and Otolaryngology wards of the Prof. Edmundo Vasconcelos Hospital, from 1985 to 2004 diagnosed with orbitary complications caused by sinusitis.

We included patients up to 12 years of age, who were analyzed according to gender, age, paranasal sinus involved, average hospital stay period, image exams performed and treatment installed.

Clinical presentation was assessed according to Chandler's classification^21^:

Group I: pre-septal cellulitis eyelid edema without palpable pus and not associated to visual loss of extra ocular mobility limitation.

Group II: orbitary cellulitis without abscess, diffuse edema of orbitary fat tissue without abscess forming.

Group III: Orbital cellulitis with subperiosteal abscess, abscess forming between the orbit periosteum and bone, eye ball shift with or without movement limitation, with or without visual acuity reduction.

Group IV: Orbital cellulitis with orbit fat tissue abscess, severe proptosis, may be frontal and not lateral or inferiorly shifting as in a subperiosteal abscess, severe limitation in eye mobility, with or without ophthalmoplegia, with or without visual loss.

Group V: Cavernous sinus thrombosis, orbital phlebitis expanding within the cavernous sinus and crossing the basilar plexus towards the other side, resulting in bilateral disease.

## RESULTS

Between 1985 and 1999 25 children diagnosed with orbit complications of acute sinusitis were seen at the Prof. Edmundo Vasconcelos Hospital (Table 1).

**Table 1:** Distribution of patients with orbitary complications according to gender.

GENDER	PATIENTS	TOTAL
MALES	17	17 (68%)
FEMALES	8	8 (32%)

Among these children, 17 were males and 8 were females, yielding an approximate ratio of 2 males for each female. Average age of these children was 6.5 years (Table 2).

**Table 2:** Patient distribution according to age range.

AGE	MALES	FEMALES
<5 YEARS	13	3
5 - 9 YEARS	1	3
9 - 12 YEARS	3	2
TOTAL	17	8

Most common complaints (24 patients) were eyelid edema and hyperemia without loss of visual acuity (Chandler's Group I). One patient presented with proptosis and reduction of ocular mobility, compatible with Chandler's Group III.

Table 3 depicts the distribution of patients in relation to age, gender and paranasal sinus involved.

**Table 3:** Case frequency by age range, according to paranasal sinus involved.

Age	Maxilary	Fronto-ethmoid	Ethmoid+ maxilary+s phenoid	Ethmoid
<5 YEARS	15			1
5-9 YEARS	2	2		
9-12 YEARS	3		1	1
TOTAL	20	2	1	2

Average hospital stay for the 25 patients was of 4 days, varying between 1 and 11 days.

As far as image exams are concerned, all 25 patients presented simple x-rays showing mucosal thickness above 4mm, air-liquid level or opacification of the involved sinus.

Of the 25 patients, 2 underwent CT scans, which showed increase in mucosal thickness, air-liquid level, and total opacification of both the frontal and ethmoid sinuses. One of them showed a subperiosteal abscess.

Table 4 lists the antibiotic agents used during hospital stay.

**Table 4:** Type of treatment installed - clinical or surgical.

TYPE OF ANTIBIOTIC	NUMBER OF PATIENTS
CRYSTALIN PENICILIN (PENI)	12
PENI + CHLORANPHENICOL (CLORA)	1
PENI + SULFA	1
PENI + OXACILIN + CHLORA	1
CHLORA + AMPICILIN	3
CEFALEXIN	3 + SINUSECTOMY
CHLORA	1
CHLORA + OXACILIN	1
LYNCOMICIN	1
CEFTRIAXONE	1
TOTAL	25

Two patients received complementary surgical treatment (endoscopic transnasal drainage of the paranasal sinuses). Moreover, the patient with subperiosteal abscess underwent external drainage. In all the cases, antimicrobial drugs were used for at least 14 days, with favorable infection outcome and full disease remission.

## DISCUSSION

Complications of acute rhinosinusitis seem to occur more frequently in children than in adults, and are directly linked to the intimate anatomical relations of the paranasal sinuses with structures of the head, neck and thorax. Although the literature reports the incidence of 7%2 in current times, our study proved it to be relatively uncommon. We believe that one of the reasons for the low incidence of such complications in our Hospital may be due to the fact that it is a private hospital. So much so that the economical and intellectual level of family members surely impact positively as far as early diagnosis and treatment are concerned.

Studies found in the literature had most of their patients below 6 years of age (50% in Fearons's studies) or below 4 years (50% in Hawkins's studies). Shahin24 revised all age groups and found 75% of the patients in the age range below 16 years and 33% below 4 years. Nonetheless, older children and adults tend to have more severe orbitary complications

Orbit cellulitis may occur after trauma or intra-orbital surgery, however, it is more frequently found in children as a complication of sinusitis, especially of ethmoidal sinusitis^3^,^5^,^12^. Although Chandler et al. described and classified orbitary infection pathogenesis in relation to acute sinusitis; we noticed that such classification does not encompass all the types of complications that may occur secondary to sinus infections, such as the osteolytic complications of the frontal sinus, or even intracranial complications. Moreover, this classification contradicts itself when in includes cavernous sinus thrombosis among orbitary complications, since the latter is really an intracranial complication. Nonetheless, since it still remains as the most used classification internationally, it is the one we adopted here.

Of our 25 patients with acute sinusitis complications, 16 cases (64%) were below 5 years of age, 4 children were between 5 and 9 years and 5 were between 9 and 12 years of age. Such results match the ones found in the medical literature, according to which this fact could be related to peculiar anatomical factors belonging to younger patients, such as the frailty of the separation between orbital content and the ethmoidal labyrinth, presence of many bone sutures (open in children) and neurovascular foramen on the medial orbitary wall, wider foramen, more porous bones, which facilitate disease dissemination and medial orbitary wall dehiscence^20^, ^21^, ^22^,^25^.

It was interesting to notice that among our cases, the incidence of complications was significantly higher in males (17-68%) when compared to females (8-32%).

In Shahin's series^24^ the ethmoid was the most commonly affected sinus in patients with orbitary complications. The maxillary sinus came in second and the frontal sinus in third. Sphenoid Involvement is rare; however, Fearon described one case of sphenoid sinusitis associated to severe central encephalopathy and visual impairment.

In all the studies present in the literature, type B Haemophilus influenza was the most commonly microorganism cultured in blood dishes^12^. Staphylococcus was the most commonly found pathogen in the secretion aspirated from the maxillary sinus (Fearon).

In our patients we cultured the secretion from the paranasal sinuses in the three cases that underwent sinusectomy and also from the material collected during surgery. There was no bacterial growth in these cultures. Such result may be related to antimicrobial treatment installed prior to surgery.

In the 2 surgery cases, the decision to operate was based on the fact that the patient was not evolving well despite clinical treatment after 24-48h, and the involvement of more that one anatomical site. Moreover, one of the patients presented one orbitary subperiosteal abscess causing proptosis and eye mobility limitation. In relation to complementary image exams, CT scan was ordered for the 2 more severe cases, thus allowing for a more precise pinpointing of the involved paranasal sinus, the orbit abscess and its extension. All patients evolved well, without sequels.

## CONCLUSION

Results from our study allow us to conclude that:

The incidence of sinusitis-related orbitary complications is not very frequent in children (6% in our series), and although most of the cases are diagnosed in their early stages, thus allowing good recovery with clinical treatment, it is a severe disease and surgery may be necessary.
